# Genome-Wide Association Studies of Growth Trait Heterosis in Crossbred Meat Rabbits

**DOI:** 10.3390/ani14142096

**Published:** 2024-07-18

**Authors:** Zhanjun Xiao, Yuchao Li, Li Yang, Mingyan Cui, Zicheng Wang, Wenqiang Sun, Jie Wang, Shiyi Chen, Songjia Lai, Xianbo Jia

**Affiliations:** 1 Farm Animal Genetic Resources Exploration and Innovation Key Laboratory of Sichuan Province, Sichuan Agricultural University, Chengdu 611130, China; xiaozj1029@163.com (Z.X.); nk695454@163.com (M.C.); 18281257677@163.com (Z.W.); wqsun2021@163.com (W.S.); chensysau@163.com (S.C.); laisj5794@163.com (S.L.); 2 College of Animal Science and Technology, Sichuan Agricultural University, Chengdu 610000, China; lichao_1116@163.com (Y.L.); 809079948@163.com (L.Y.); wjie68@163.com (J.W.)

**Keywords:** meat rabbit, GWAS, ROH, SNP, heterosis

## Abstract

**Simple Summary:**

The application of advantages can not only effectively improve the disease resistance and meat quality of livestock, but also significantly promote the reproduction and growth of livestock and poultry. A total of 78,579 SNPs and 42,018 ROHs were detected from autosomal samples of 380 meat rabbits after quality control for whole-gene association analysis using a mixed linear model. The homozygosity of the population genome was evaluated, and the number, length, frequency, and distribution of ROHs in the population were analyzed. Notably, candidate genes associated with growth and development were found in the high-frequency ROH region. In this study, the identified candidate genes can be used as molecular markers for assisted selection in meat rabbits. At the same time, the inbreeding situation based on ROH evaluation can provide reference for breeding and breeding preservation of meat rabbits.

**Abstract:**

The application of heterosis can not only effectively improve the disease resistance and meat quality of livestock, but also significantly enhance the reproduction and growth of livestock and poultry. We conducted genome-wide association studies using data from F2 crossbred meat rabbits to screen out candidate genes with significant dominant effects associated with economic trait variation. High-throughput sequencing technology was used to obtain SNPs covering the whole genome to evaluate the homozygosity of the population genome, and analyze the number, length, frequency, and distribution of ROHs in the population. Candidate genes related to economic traits of meat rabbits were searched based on high-frequency ROH regions. After quality control filtering of genotype data, 380 F2 crossbred rabbits were identified with 78,579 SNPs and 42,018 ROHs on the autosomes. The fitting of the Logistic growth curve model showed that 49-day-old rabbits were a growth inflection point. Then, through genome-wide association studies, 10 SNP loci and seven growth trait candidate genes were found to be significantly related to body weight in meat rabbits at 84 days of age. In addition, we revealed the functional roles and locations of 20 candidate genes in the high-frequency ROH region associated with economic traits in meat rabbits. This study identified potential genes associated with growth and development in the high-frequency ROH region of meat rabbits. In this study, the identified candidate genes can be used as molecular markers for assisted selection in meat rabbits. At the same time, the inbreeding situation based on ROH assessment can provide reference for breeding and breeding preservation of meat rabbits.

## 1. Introduction

Rabbit meat is a nutritious meat that is rich in potassium, phosphorus, selenium, and B vitamins [1,2]. Its protein content is much higher than other meats, and its fat and cholesterol content is lower than that of other livestock and poultry meats [3,4]. In addition, the digestibility of rabbit meat is high, and it can be easily to be digested and absorbed after consumption, making it an ideal choice for obese people and cardiovascular patients. Growth traits are important economic traits in meat rabbit breeding. Screening candidate genes related to heterosis of growth traits in meat rabbits is very important to improve the production efficiency of commercial meat rabbits and obtain high yield, high quality, and low-cost commercial rabbits.

Genome-wide association studies (GWAS) represent a statistical approach based on the principle of linkage disequilibrium (LD) that uses molecular marker technology to conduct statistical studies of genetic variation across the entire genome to identify genetic markers associated with target traits. GWAS technology was first proposed by Risch [5]. Subsequently, Klein et al. [6] found important genetic factors of age-related macular variation through GWAS analyses in 2005. This marked the official beginning of a flurry of research into the use of a GWAS to reveal the genetic basis of complex traits. Moreover, the epigenome comprising different mechanisms, e.g., DNA methylation, remodeling, histone tail modifications, chromatin microRNAs, and long non-coding RNAs, interacts with environmental factors like nutrition, pathogens, and climate to influence the expression profile of genes and the emergence of specific phenotypes [7]. Multi-level interactions between the genome, epigenome, and environmental factors might occur. Furthermore, numerous lines of evidence suggest the influence of epigenome variation on health and production. The expression of eukaryotic genes is temporally and multidimensionally controlled. Only a relatively small set of the entire genome is expressed in each type of tissue, and the expression of genes depends on the stage of development. Therefore, gene expression in eukaryotes is specific to each tissue [8]. Also, the amount of gene products that are made in the same tissue as well as in other tissues that make up that product regulates the expression of that gene. One of the basic activities in domestic animals is the study of genes and proteins related to economic traits and their study at the cellular or chromosomal level [9].

A run of homozygosity (ROH) refers to the same allele genes obtained by offspring from their two parents and can be used to identify the degree of inbreeding [10,11,12,13]. Genetic drift, artificial selection, natural selection, population bottleneck, mutation rate, linkage imbalance, and inbreeding all affect the generation of ROHs [14,15,16]. The average length of the ROHs as well as the coverage information in the genome can be used to infer the genetic history of the population [17]. By measuring the length of the ROH, it is possible to infer the distance between an individual and their common ancestor. The longer the ROH is, the closer the generation distance between the two is. Conversely, the shorter the ROH is, the more distant they are [18]. The proportion of the total length of all ROH to the entire length of the genome is used to represent the inbreeding coefficient (F_ROH_) [19]. The ROH analysis is a complement to the study of inbreeding in a GWAS, and provides convenience for using SNP information to conduct animal kinship identification [17]. Currently, crossbreeding methods are widely used in rabbit meat production, but there is a lack of comprehensive research on crossbreeding. In recent years, the use of genomic data to study the coefficient of kinship and population selective traits in the rabbit industry has become increasingly common [20,21,22]. Therefore, studying the genetic history of populations and inbreeding in ROHs will contribute to subsequent breeding, thereby further improving the heterosis of economic traits in meat rabbits [21].

The original sequencing data and SNP calling used in the experiment were from previous studies of the research group [23]. In this experiment, crossbred offspring of Kangda 5 (K5) rabbits and California (CA) rabbits were used as the research subjects. The high frequency range of ROHs was determined through ROH analysis and statistics. Gene annotation and enrichment analysis on SNP in the high-frequency region of ROHs were performed to identify genes related to economic traits of meat rabbits, which include their growth and development.

## 2. Materials and Methods

### 2.1. Animals and Genotypes

The experiment was conducted at the rabbit breeding farm of Kangda Group Co., Ltd. (Qingdao, China). All experimental rabbits were raised at a density of two per cage/layer from 35 to 70 days of age, and single-caged after 70 days. The breeding environment maintained a temperature range of 18–25 degrees and a humidity of 20–70%. From the breeding rabbit population, 30 healthy 5.5-month-old CA female rabbits and 15 K5 series male rabbits were randomly selected as the parents for the resource group. Light treatment was used to induce synchronous estrus, and natural mating was employed to obtain F1 generation rabbits (129 in total, 65 female rabbits and 64 male rabbits) that met the breeding requirements. The non-consanguineous F1 generation rabbits were randomly mated to produce 432 F2 generation individuals (200 female rabbits and 232 male rabbits). The original sequencing data and SNP calling used in the experiment were from previous studies of the research group [23], and the genotype data used in this experiment only include autosomal SNPs.

Based on the original data, we carried out systematic quality control of the original genotype data by referring to Liao’s method [24], and remove unqualified samples. Use GATK v 4.2 [25] to set the parameter “QD < 2.0||FS > 60.0||MQ < 40.0” to filter SNP. To obtain high quality SNPs, we first used Ubuntu v 20.24 (https://releases.ubuntu.com/focal/, (accessed on 20 December 2021)) to screen for heterozygotes. Then, we used PLINK v.1.9 [26] run-with the following parameters for quality control: (i) SNP call rate—0.9; (ii) Sample call rate—0.8; (iii) Minor allele frequency—0.05; (iv) Hardy—Weinberg equilibrium—1 × 10^−6^. After filtering and populating the genotype data according to the quality control criteria, we obtained a total of 78,579 SNPs on the autosomes of 380 F2 generation crossbred rabbits (205 male rabbits and 175 female rabbits).

### 2.2. ROH Calling and Inbreeding Coefficients

The final dataset contained genotypes at 78,579 SNPs autosomal SNPs for 380 individuals for which annual survival data were available. Calculate all ROHs for all individuals using the --homozyg function in PLINK [27], and the following parameters:

--homozyg-gap 100\

--homozyg-density 50\

--homozyg-kb 300\

--homozyg-snp 50\

--homozyg-window-snp 50\

--homozyg-window-het 3\

--homozyg-window-threshold 0.05\

--out ${output}

For the shorter ROH, it reflects lower inter-individual variability and greater generational distance. Therefore, we chose 300 kb as the minimum ROH length to explore the correlation between ROHs and heterosis. We refer to the length classification of ROHs by Schiavo and Ferenčaković et al. [10,28], and use the same formula to classify the identified ROHs into three categories: ROH 0.3–2 (ROH ≥ 0.3 Mb and <2 Mb); ROH2–4 (ROH ≥ 2 Mb and <4 Mb); ROH > 4 (ROH ≥ 4 Mb). The population genomic inbreeding was evaluated by descriptive statistics of the number, length, and distribution frequency of ROHs. We calculated the individual F_ROH_ by summing the total ROH length for each individual and dividing it by the total autosomal genome length [29]. For the obtained high-quality SNPs, the sample’s kinship was computed using MEGA5 v. 11.0.10 [30] (neighbor-joining algorithm) software.

### 2.3. Estimation of Individual Heterosis at Different Growth Time Points

A total of 380 crossbred rabbits retained after quality control were used to assess the strengths of seven different growth stages. The survival data at seven time points of 35, 42, 49, 56, 63, 70, and 84 days of age were fitted with the Logistic nonlinear animal growth curve model [26] to obtain the weight of experimental rabbits at 35~84 days of age:

$$W=A\times {\left[(1-B\times e\left(-k\times t\right)\right]}^{3}$$

where A is the limiting growth amount; k is the instantaneous relative growth rate; and B is the constant scale. The inflection point weight is derived from A/2; (InB)/k is the turning point day age.

Heterosis and daily gain heterosis were defined for each individual by the following expression:

$$\mathrm{dw}=\frac{\left(d2-d1\right)}{t}$$

$$H=\frac{F2-\left(P1+P2\right)/2}{\left(P1+P2\right)/2}\times 100\%$$

where dw is daily gain; d2 − d1 is the weight of a certain period of time; t is day of age, H is heterosis; F2 is the individual weight of the F2 generation; and P1 and P2 are the natural weight of the parents of the F2 generation.

### 2.4. Heterosis Evaluation of Growth Traits

We used the mixed linear model (MLM) proposed by Akanno [31] to analyze the correlation between SNPs obtained and heterosis of growth traits:

yit=xb+wδ+e

where yit is the weight of the animal at day age; b is for fixed effect, which consists of (sex) and (principal component; x is the association matrix of b; w is the dominant effect vector of each SNP, the heterozygous genotype (AB) is encoded as 1, and the two homozygous genotypes (AA and BB) are encoded as 0. δ is the dominant effect; e is a random residual effect vector.

We used the SnpEff v. 5.0-0 to obtain the positions of SNPs significantly associated with the target trait, as well as the relative positions of nearby genes. Annotate the SNPs using the rabbit reference genome OryCun2.0 (https://www.ncbi.nlm.njyih.gov/genome/?term=rabbit, (accessed on 20 December 2021)) and its corresponding genome annotation information, and then the strength of linkage disequilibrium (LD) among SNPs was calculated. Based on the results of linkage disequilibrium analysis, use the BioMart package of R v. 4.2.1 (https://www.r-project.org/ (accessed on 20 December 2021)) to extract genes within 1 Mb upstream and downstream of the SNP sites significantly associated with the target trait [32], and select them as candidate genes associated with the trait. Functional analysis of the candidate genes was performed, including Gene Ontology (GO) and Kyoto Encyclopedia of Genes and Genomes (KEGG) enrichment analysis, through annotation, visualization, and integration with the DAVID bioinformatics resources (available online: https://david.ncifcrf.gov/ (accessed on 20 December 2021)). A corrected *p* < 0.05 was considered significant enrichment.

## 3. Results

### 3.1. Phenotypic Data Analysis

**Figure 1:**
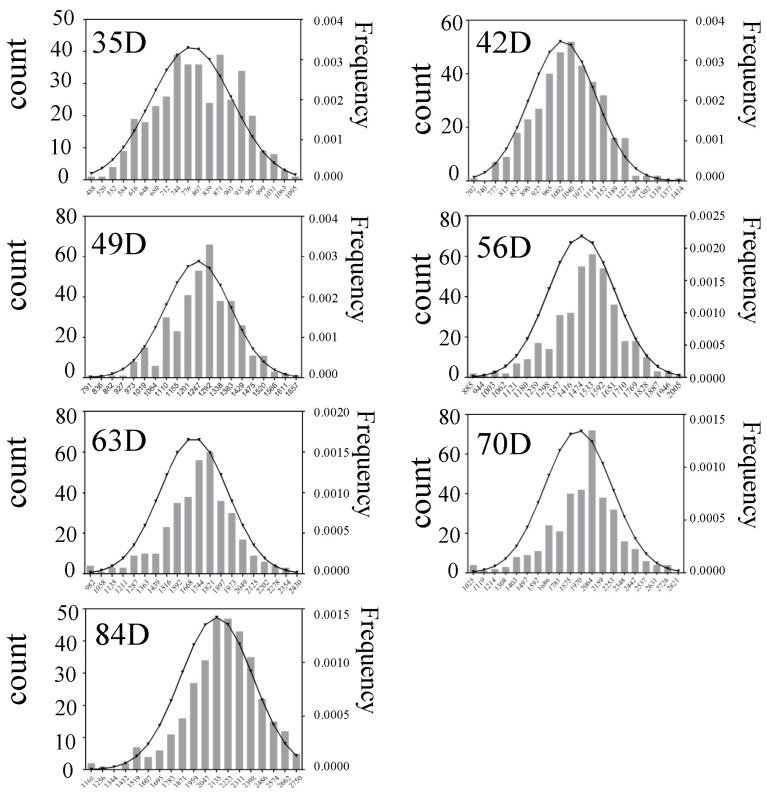
The weight frequency distribution of 380 samples of F2 generation rabbits at seven growth stages (35, 42, 49, 56, 63, 70, and 84 days old). Note: The histogram represents the frequency distribution, and the curve represents the normal distribution curve.

After quality control of the phenotype data of the F2 generation group with the Liao method [26], a total of 380 rabbits in the F2 generation (205 male rabbits and 175 female rabbits) were obtained for genotyping and data analysis. The phenotypic values and advantages of 380 valid samples retained after quality control in seven growth periods were described. The statistics included average body weight at 35, 42, 49, 56, 63, 70, and 84 days of age is shown in Table 1. Average advantage of each group is shown in Table 2. In addition, average daily weight gain and heterosis from days 35 to 42, 42 to 49, 49 to 56, 56 to 63, 63 to 70, and 70 to 84 are shown in Table 3. As shown in Figure 1, the weights of the 380 valid samples in F2 generation were normally distributed across all seven growth stages.

### 3.2. Genotyping and Population Stratification

**Figure 2:**
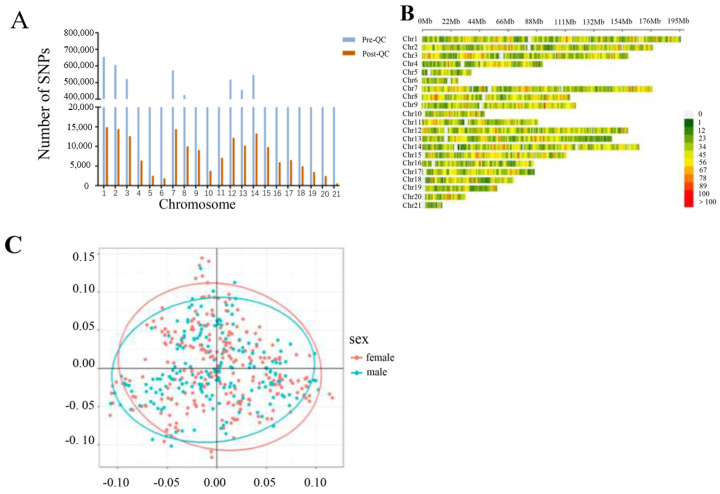
(**A**) The number of SNPs on each autosome before and after quality control. (**B**) Autosome SNP density distribution plot. (**C**) Principal component analysis of SNPs.

The number of SNPs on each chromosome changed significantly before and after QC, as shown in Figure 2A. After screening and imputation of genotype data according to QC standards, a total of 78,579 SNPs were retained on the autosomes of 380 samples (205 male rabbits and 175 female rabbits). The distribution of SNPs on the 21 autosomes is shown in Figure 2B. The effective number (N), average distance (AD), and minor allele frequency (MAF) of SNPs on each autosome were also calculated or assessed (Table 4).

The presence of population stratification may lead to changes in allele frequencies, resulting in subpopulations. This change may affect the accuracy of the GWAS, leading to false positive results [33]. Based on the effective SNPs obtained after quality control, principal component analysis was performed using PLINK v1.9 [26] (Figure 2C), and the results were analyzed using PCA for R v. 4.2.1 (https://www.r-project.org/) (accessed on 20 December 2021). The results indicate no apparent stratification among samples.

### 3.3. Correlation Analysis of Growth Traits

**Figure 3:**
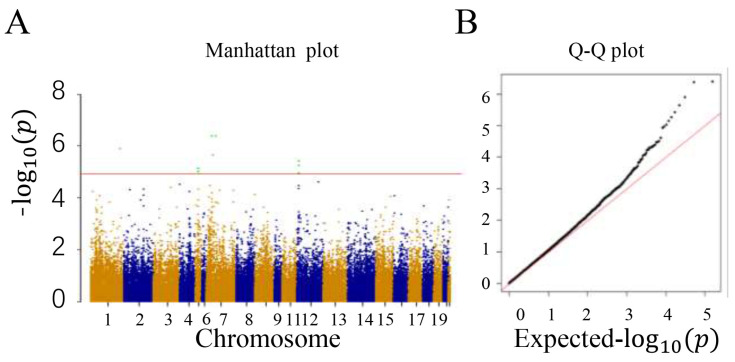
84-day old GWAS Manhattan chart and Q-Q chart. The X-axis in (**A**) is color-coded to represent different chromosomes, and the Y-axis represents the −log10(*p*) of the SNPs. The horizontal red line parallel to the *X*-axis represents the genome-wide significance level threshold. (**B**) Sites on the red line indicate genome-wide significance and are associated with target traits.

The experiment used the MLM model to perform a GWAS of phenotypes and quality-controlled 78,579 SNPs in 380 samples. A total of 10 SNPs reaching a significant level of FDR (*p* < 0.01) were detected. These significant SNPs were all significantly associated with body weight at 84 days and were distributed on chromosomes 1, 5, 7, and 12. Annotation within 1 Mb upstream and downstream of all significant SNPs identified a total of 99 candidate genes (Supplementary Table S1), including 85 protein-coding genes and 14 long non-coding RNAs. Through the localization of candidate genes (Figure 3), *ABTB2*, *CDH11*, *CNTNAP5*, *PPP1R3A*, *JARID2*, *GPX5*, and *GPX6* were discovered. Ultimately, we believe that these seven genes may be candidate genes affecting the heterosis of meat rabbit growth traits.

Functional analysis of candidate genes found no relevant functional descriptions for the 14 long non-coding RNAs. Among the 85 protein-coding genes, 21 are novel genes (Supplementary Table S1). Enrichment analysis identified 22 GO entries related to biological processes and six KEGG pathways (Supplementary Tables S2 and S3). Among them, 18 GO entries and five KEGG pathways reached significant levels (corrected *p* < 0.05 was considered significantly enriched). Both candidate genes *CAPRIN1* and *JARID2* were involved in the most significant and highly enriched GO entry: gene expression. In addition, candidate gene *JARID2* is involved in nine biological processes, such as the cellular macromolecule biosynthetic process and gene expression process.

### 3.4. ROH and Correlation of Inbreeding Coefficients

**Figure 4:**
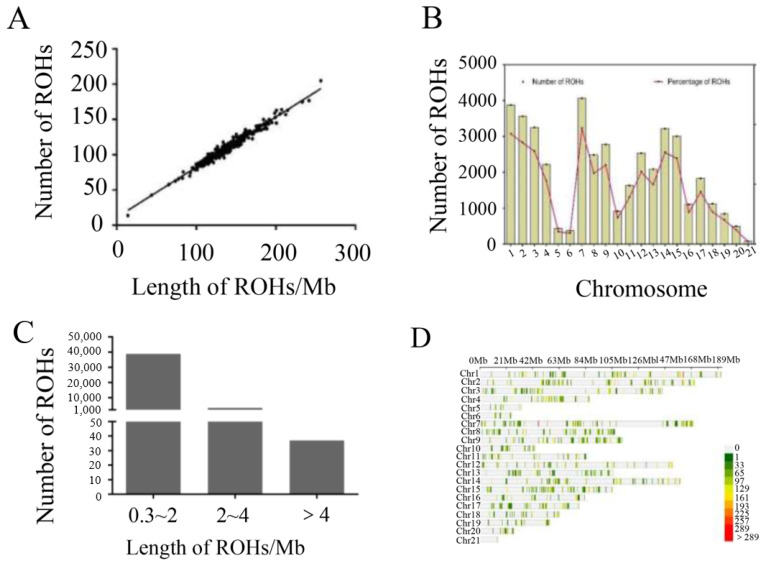
(**A**) Scatterplot of total length of ROH per individual within the population. (**B**) Histogram of the number of short, medium, and long fragments of ROH. (**C**) Density distribution of ROHs. The shorter ROH segments (0.3~2 Mb) account for a high proportion. (**D**) Histogram of ROH distribution for different chromosomes.

In the F2 generation, a total of 42,018 ROHs were detected from 380 samples. The quantity of ROHs is highly correlated with its total length, ranging from 0.9757 to 0.9837 (Figure 4A). Based on the total length of ROHs, we classified ROHs into three categories: 0.3~2 Mb, 2~4 Mb, and >4 Mb [34,35]. The shorter ROH segments (0.3~2 Mb) account for a high proportion (Figure 4B), with an average length of 0.93Mb. The ROH on each chromosome were visualized (Figure 4C,D) and the number, average length, and inbreeding coefficient of the ROHs were calculated (Table 5).

We used four meat rabbit fitting curve models established by Liao [26], the Logistic, Gompertz, Brody, and Von Bertalanffy, all of which successfully fit the growth curve of the F2 generation sample. It was found that the Logistic model had the best fitting effect, with a high model fitness of 0.996. Therefore, in this experiment, we fitted the growth curve of the population based on the Logistic model. The fitting results of the growth curve indicate that the inflection point of growth is 49 days old. Hence, the analysis of ROHs and F_ROH_ at 49 days old shows that the range of correlation with heterosis is −0.1267 to 0.07436 for ROHs and 0.8754 to 0.9150 for ROHs and F_ROH_.

### 3.5. Functional Annotation of Candidate Genes in ROHs

**Figure 5:**
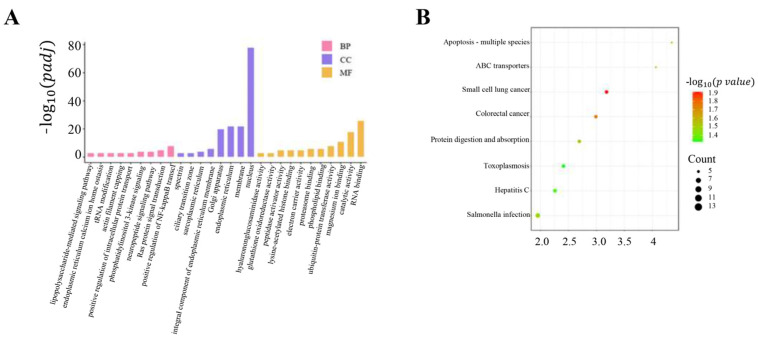
(**A**) Twenty-eight Gene Ontology entries (BP: biological process; CC: cellular component; MF: molecular function). (**B**) Eight important KEGG pathways.

Having performed frequency statistics on SNPs of ROHs, the top 5% high-frequency SNPs were defined as high-frequency SNPs, and the ROH regions formed by them were defined as high-frequency ROH regions (a total of 1924 were detected). Annotation of SNPs in high-frequency regions identified 1018 candidate genes, including 748 protein-coding genes and 270 long non-coding RNAs (Supplementary Table S4). By annotating the genes in the high frequency region and using the whole-genome SNPs’ marker information, 20 genes related to economic traits were identified. (Table 6). GO functional and KEGG signaling pathway enrichment analysis was performed on the selected candidate genes. The results indicated 28 GO entries, including nine biological processes, eight cellular components, and 11 molecular functions, were closely related, as shown in Figure 5A. The most important biological processes were catalytic activity, magnesium ion binding, and ubiquitin-protein transferase activity, particularly those related to RNA binding. Additionally, eight significant KEGG signaling pathways are shown in Figure 5B. *MAP2K6* and *MEP1B* are involved in the signaling pathways of Salmonella infection and protein digestion and absorption, respectively (Supplementary Table S5).

## 4. Discussion

An ROH is a continuous segment of homozygous genotype in the diploid organism genome, formed by the same allele inherited from the parents by the offspring. The genetic relationship and their common ancestor can be inferred by measuring the length of the ROH. The distribution of ROH length classes can provide insights into population history and inbreeding level [14,35]. Previous studies have divided the length of ROHs into four intervals: >1, >2, >8, and >16 Mb, corresponding to the common ancestors of 50, 25, six, and three generations, respectively [10]. Long ROH fragments reflect inbreeding in recent generations, while a short ROH indicates inbreeding in more distant generations. In this study, we conducted ROH analysis on the phenotype and genotype data of seven different growth stages of F2 generation 380 crossbred rabbits. The results show that short ROH segments account for a large proportion (72.29%) in the research population, with an average size of 0.93 Mb. Due to the lower breeding level of local breeds compared to commercial breeds, the proportion of short ROH segments in local breeds was found to be higher than that in commercial breeds [34,36,37]. The two parental rabbit populations in this study had a low probability of inbreeding, which is consistent with previous research.

Inbreeding produces offspring that may lose certain characteristics compared to their parents [38,39]. However, it also affects the length and quantity of ROHs. Therefore, ROH is often used to assess the degree of inbreeding in livestock populations [40]. Then, we add up the total length of ROHs for each individual and divide it by the total length of the autosomal genome to calculate the individual inbreeding coefficient (F_ROH_). The correlation between ROHs and F_ROH_ is also explored. The results show a high correlation between ROHs and F_ROH_. In previous studies of the Jianchang black goat [41], there was a strong positive correlation between the number of ROHs on each chromosome and the chromosome length. Similar results were also found in studies of the German white cashmere goat [42]. Individual inbreeding coefficients calculated using ROHs based on genomic information will be more accurate and comprehensive. Annotation of SNPs in the high-frequency region of ROHs is also conducted to identify potential genes related to growth, development, and adaptability.

This study identified seven candidate genes that may affect the heterosis of meat rabbit growth traits, including genes *CDH11*, *JARID2*, *ABTB2*, *CNTNAP5*, *PPP1R3A*, *GPX5*, and *GPX6*. It has been reported that *CDH11* is highly expressed in chicken osteoblast cell lines, which not only contributes to bone growth and development, but also plays a role in maintaining bone health [43]. In addition, targeted inhibition of *CDH11* gene expression can inhibit the proliferation of myoblasts and promote the differentiation of myoblasts. Based on GWAS analysis of beef cattle populations, *CDH11* gene expression levels may have a significant impact on cattle height and body size [44]. *GPX5* is specifically expressed in mammalian epididymis and plays an important role in protecting sperm from lipid peroxidation damage and also affects fertility [45]. In addition, when testicular dysplasia occurs, sperm concentration decreases, sperm morphological changes increase, and spermatozoa symptoms affect the economic loss of male animals and reduce the efficiency of meat production.

The association between genes related to economic traits of meat rabbits and ROHs has reached identification significance in the high-frequency ROH region. In conclusion, the results of this study identified the genetic markers existing in crossbred breeds, provided important insights for the utilization, selection, and improvement of crossbred rabbits crossbred, and provided an important theoretical basis for the development of marker-assisted selection and genome selection on growth and reproductive traits of domestic rabbits.

## 5. Conclusions

This study identified 380 samples of F2 generation crossbred rabbits based on phenotype data selection. A GWAS of 78,579 SNPs was conducted, and ROHs were detected. We also used the ROHs to study the F_ROH_ of meat rabbits and determined that a large proportion of short ROH segments are present in the current inbred meat rabbit population. Additionally, the number of ROHs on each chromosome is positively correlated with chromosome length, and there is a strong correlation between ROHs and F_ROH_. We annotated SNPs located in high-frequency ROH regions and identified potential genes related to the growth, development, and adaptability of meat rabbits. This may be due to the heterosis exhibited by the crossbred population, which combines the advantages of both parents. In summary, the results of this study provide an important basis for screening candidate genes related to heterosis of growth traits in meat rabbits, and provide an important theoretical basis for carrying out marker-assisted selection and genome selection for growth traits in crossbred meat rabbits.

## Figures and Tables

**Table 1:** F2 generation weight at seven growth time points after quality control.

DA	N	Max1 (g)	Min1 (g)	M1 (g)	SD (g)
35	378	1095	456	786.31	120.42
42	379	1414	665	1011.42	114.37
49	380	1657	745	1243.56	138.14
56	380	2005	826	1473.96	182.26
63	360	2354	906	1706.34	238.28
70	352	2726	930	1943.69	295.90
84	339	2750	1080	2136.16	280.64

DA: days of age; N: the number of valid samples; Max1: maximum body weight; Min1: minimum body weight; M1: mean weight; SD: standard deviation.

**Table 2:** Average advantage at seven growth time points after quality control in F2 generation.

DA	N	Max2 (%)	Min2 (%)	M2 (%)	SD (%)
35	378	0.485	−0.378	−0.006	0.176
42	379	0.418	−0.323	−0.012	0.133
49	380	0.259	−0.492	−0.119	0.124
56	378	0.463	−0.506	−0.055	0.146
63	358	0.514	−0.517	−0.016	0.155
70	345	0.370	−0.548	−0.011	0.161
84	332	0.306	−0.523	−0.017	0.139

DA: days of age; N: the number of samples; Max2: maximum heterosis value; Min2: minimum heterosis value; M2: mean heterosis; SD: standard deviation.

**Table 3:** Daily gain and daily gain heterosis in adjacent growth stages of F2 generation.

Character	35~42 DA	42~49 DA	49~56 DA	56~63 DA	63~70 DA	70~84 DA
DG	31.91 ± 10.63	32.99 ± 9.21	32.91 ± 9.35	32.91 ± 9.35	33.02 ± 9.56	12.06 ± 23.95
HW	0.001 ± 0.39	−0.37 ± 0.28	−0.39 ± 4.23	0.78 ± 2.16	0.14 ± 0.59	−0.13 ± 2.32

DG: the daily gain of weight; HW: the heterosis of weight for day age.

**Table 4:** The effective number, average distance, and minor allele frequency of SNPs on each autosome.

Chromosomes	N	AD (bp)	MAF
1	6966	27.966	0.264
2	6654	26.137	0.257
3	5894	26.383	0.251
4	3205	28.495	0.238
5	1195	31.161	0.268
6	817	33.428	0.239
7	6752	25.705	0.249
8	4714	23.620	0.252
9	4613	25.090	0.254
10	1769	25.160	0.248
11	3278	26.673	0.248
12	5669	27.375	0.252
13	4849	29.524	0.261
14	6000	27.300	0.259
15	4517	23.464	0.252
16	2809	30.064	0.259
17	3207	26.484	0.269
18	2450	27.053	0.249
19	1748	30.828	0.249
20	1178	25.569	0.243
21	295	42.013	0.244

N: the effective number; AD: average distance; MAF: minor allele frequency.

**Table 5:** T Mean length and number of ROHs and genome-wide ROH-based inbreeding coefficients.

Chromosomes	Samples	ROHs	Qty	Avg (Mb)	F_ROH_
1	380	3878	171	1.24 ± 0.46	7.01 × 10^−8^
2	380	3567	193	1.32 ± 0.51	8.04 × 10^−8^
3	379	3257	91	1.22 ± 0.43	8.83 × 10^−8^
4	378	2223	149	1.36 ± 0.63	1.92 × 10^−7^
5	209	443	0	1.06 ± 0.27	1.90 × 10^−7^
6	255	385	17	1.50 ± 0.44	5.22 × 10^−7^
7	380	4065	281	1.27 ± 0.66	8.73 × 10^−8^
8	379	2489	38	1.22 ± 0.41	1.13 × 10^−7^
9	381	2781	197	1.35 ± 0.56	1.29 × 10^−7^
10	326	921	59	1.20 ± 0.55	2.41 × 10^−7^
11	377	1638	2	1.15 ± 0.37	1.33 × 10^−7^
12	380	2539	101	1.23 ± 0.46	7.03 × 10^−8^
13	375	2090	132	1.38 ± 0.51	8.73 × 10^−8^
14	380	3221	79	1.19 ± 0.41	7.32 × 10^−8^
15	380	3009	119	1.25 ± 0.51	1.47 × 10^−7^
16	353	1109	124	1.31 ± 0.63	1.16 × 10^−7^
17	379	1835	111	1.32 ± 0.57	1.73 × 10^−7^
18	358	1131	0	1.00 ± 0.28	1.24 × 10^−7^
19	349	849	56	1.28 ± 0.54	2.05 × 10^−7^
20	269	501	3	1.25 ± 0.38	2.82 × 10^−7^
21	87	87	0	0.89 ± 0.05	2.75 × 10^−7^

Qty: the quantity of the high-frequency ROH regions; Avg: average length of ROHs.

**Table 6:** Location information of candidate genes related to economic traits.

Chromosome	Significant SNPs	Position (bp)	Candidate Genes
1	ENSOCUG00000014693	115177017	*PGR*
2	ENSOCUG00000002594	62292147	*SLC25A4*
	ENSOCUG00000011227	135606186	*LHCGR*
	ENSOCUG00000010418	49018842	*SAP30*
	ENSOCUG00000009784	135741577	*FOXN2*
4	ENSOCUG00000017314	63287210	*MGAT4C*
	ENSOCUG00000005909	44797807	*HELB*
6	ENSOCUG00000001191	11540770	*LYRM1*
7	ENSOCUG00000011449	19355249	*SPAM1*
	ENSOCUG00000012455	29470957	*MDFIC*
	ENSOCUG00000000303	29470957	*PPP1R3A*
9	ENSOCUG00000013743	72501512	*MEP1B*
10	ENSOCUG00000015979	16510779	*DPY19L1*
13	ENSOCUG00000012320	44937297	*TBX15*
14	ENSOCUG00000001739	129651182	*CADM2*
15	ENSOCUG00000008959	94164303	*MYOZ2*
	ENSOCUG00000001300	57092972	*CCSER1*
16	ENSOCUG00000006634	17521122	*FAM107B*
17	ENSOCUG00000011430	49383678	*AP4S1*
19	ENSOCUG00000007606	52334275	*MAP2K6*

## Data Availability

The datasets supporting the conclusions of this article are included within the article and Supplementary Materials.

## Supplementary Materials

The following supporting information can be downloaded at: https://www.mdpi.com/article/10.3390/ani14142096/s1, Table S1: A total of 85 protein-coding genes were significantly associated with weight heterosis at 84 days of age; Table S2: GO analysis of heterosis candidate gene; Table S3: Heterosis correlation of KEGG enrichment analysis; Table S4: GO analysis of genes in the high-frequency ROH region; Table S5: KEGG enrichment analysis of candidate genes in high-frequency ROH region.
